# Radiographic determination of cardiomegaly using cardiothoracic ratio and transverse cardiac diameter: can one size fit all? Part one

**DOI:** 10.11604/pamj.2017.27.201.12017

**Published:** 2017-07-14

**Authors:** Edmund Kwadwo Kwakye Brakohiapa, Benard Ohene Botwe, Benjamin Dabo Sarkodie, Eric Kwesi Ofori, Jerry Coleman

**Affiliations:** 1 University of Ghana School of Medicine and Dentistry, Ghana; 2 Department of Radiography, University of Ghana School of Allied Health Sciences, Ghana

**Keywords:** Cardiothoracic ratio, transverse cardiac diameter, cardiomegaly

## Abstract

**Introduction:**

The cardio-thoracic ratio (CTR) and the transverse cardiac diameter (TCD) on Plain chest radiography are the two parameters commonly used to diagnose cardiomegaly and heart disease. A CTR of greater than 50% on a PA film is abnormal and normally indicates cardiac or pericardial disease condition, whiles an increase of TCD from 1.5 to 2cm on two consecutive radiographs, taken at short interval, suggests possible cardiac pathology. The aim was to determine the suitability of using the same TCD and CTR to detect cardiomegaly for all age groups and genders respectively.

**Methods:**

A retrospective study involved the review of 1047 radiological images of adults aged 21 to 80 years, who had plain postero-anterior chest radiographs between January 2012 and November 2013 by 3 radiologists. Data recorded included the transverse cardiac, thoracic diameter and the cardiothoracic ratios. Descriptive analyses were carried out using the Microsoft excel 2010.

**Results:**

The mean age and standard deviation for the study population was 35.1 ± 12.7. The mean and standard deviations for the transverse cardiac diameter, thoracic diameter, and the cardiothoracic ratios for male participants were 13.08cm ± 1.2, 29.7cm ± 2.7 and 46.6% ± 3.9; and 12.9 cm ± 1.3, 27.1 cm ± 2.6, and 47.8% ± 4.8 for females. An increase in TCD of 1cm resulted in a CTR of greater than 50.0% in all but the males aged 21-40 years.

**Conclusion:**

The study found that the same TCD and CTR values are not suitable in detecting cardiomegaly for all age groups and genders.

## Introduction

The postero-anterior (PA) chest radiograph is a common and non-invasive way to radiologically assess the size of the heart for disease and patient response to treatment [1]. There are generally three methods for assessing the size of the heart on a plain chest radiograph. The two most commonly used are by the measurement of the cardio-thoracic ratio (CTR), and the transverse cardiac diameter (TCD) [2, 3]. Different literatures suggest different cut-off points for CTR and TCD for the determination of cardiomegaly, with different editions of some books quoting different values for the same parameter [2–5]. Some Western publications, have stated normal upper limits for TCD of 13.5cm and 12.5cm, 16.0cm and 15.0cm, and 15.5cm and 14.5cm, for males and females respectively [2, 5, 6]. An increase of 1.5 to 2cm on two consecutive radiographs, taken at short interval, suggests possible cardiac pathology [5, 6].

Publications from the 1980s suggest that though the upper limit for CTR measurement is generally considered to be 50%, a ratio of 55% may be considered normal for Blacks and Asians [2, 5–7]. Neonates and the elderly may also have normal cardiothoracic ratios of up to 60% [5]. The Radiology Review Manual quotes less than 45% as being normal, 45% to 55% as mild cardiomegaly, greater than 55% as moderate to severe cardiomegaly [8]. TCD values of 16.5cm for males and 15.0cm for females, and CTR of 55.0% for both sexes were obtained in a British study by Kabala and Wilde [9]. These provided useful upper limits for discriminating between normal and abnormal heart sizes [9]. Nonetheless, the above findings have related largely to European samples with limited focus on the black population. Local studies on Ghanaian patients may help to define the TCD and CTR among the population. The use of inaccurate TCD and CTR values may result in delayed or unwarranted diagnosis if used for referencing. Delay in treatment may occur when the reference values in use are significantly greater than the actual local values usually recorded. Unwarranted medication may be administered in circumstances when actual local values are larger than figures being used. This study aimed to determine the suitability of using the same TCD and CTR to detect cardiomegaly for all age groups and genders respectively.

## Methods

**Figure 1:**
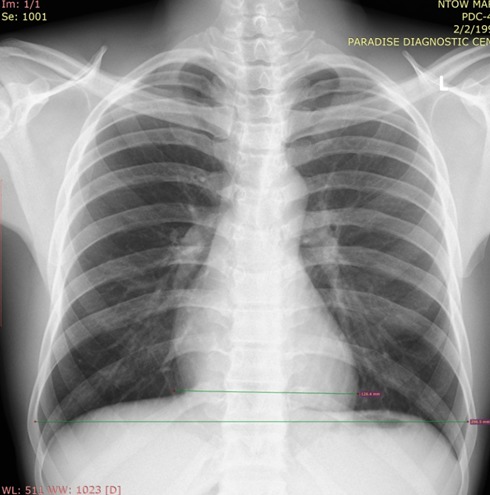
Plain chest radiograph demonstrating transverse thoracic diameter and transverse cardiac diameter measurements in a normal patient

**Figure 2:**
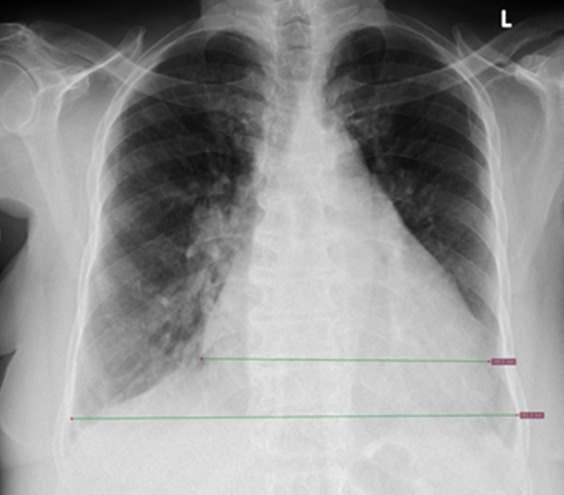
Plain chest radiograph demonstrating transverse thoracic diameter and transverse cardiac diameter measurements in a patient with cardiomegaly

A retrospective descriptive study was conducted by evaluating the radiology reports of 104^7^ individuals who had had plain postero-anterior chest radiographs between January 2012 and November 2013. The radiographs were reviewed by 3 radiologists with 5 - 10 years of post-qualification experience at 2 radiological imaging facilities in Accra, utilizing both digital and analogue radiographic equipment. The individuals included in the study were adults aged 21 to 80 years, presenting for pre-employment, pre-admission or visa medical examinations, without a history of known cardiac, or other medical disease. Radiographs were taken with a focus-to-detector distance of approximately 1.8m, and individuals standing erect in the postero-anterior position. Film exposures were made during an inspiratory breath-hold, with rib position on the 6th rib anteriorly or the 10th rib posteriorly. Data recorded included the sex, the TCD, the TTD and the CTR, the latter was derived from the formula CTR =(TCD/TTD)X 100%. The TTD was measured using the method in the Jamaican study by Ashcroft and Miall [10], which measured the inner diameter of the thorax at its widest diameter (Figure 1 and Figure 2). The equipment used comprised a digital Oldelft Benelux digital x-ray equipment (manufactured in September 2009, Germany), and an analogue LXT-20 portable x-ray machine and film processor (manufactured in July 2008, China). Data handling was by descriptive analyses. This was carried out using the Microsoft excel 2010. Ethical clearance was sought and obtained from the University of Ghana College of Health Sciences Ethical and protocol Review Committee.

## Results

A total of 1047 radiological images were reviewed. Table 1, shows the mean and standard deviation of cardiac size parameters among the various age groups. The average mean TCD, CTR, TTD and their standard deviations were 13.8cm ± 1.2, 46.6% ± 3.9, 29.7cm ± 2.7 for males, and 12.9cm ± 1.3, 49.6% ± 4.8, 27.1cm ± 2.6 for females. TCD in males increased from the 21-40 years group (13.6cm ± 1.2) to the 41-60 years group (14.4cm ± 1.1), then decreased in the above 60 years group (13.7cm ± 1.2). In females, TDC increased from the 21 - 40 years group (12.5cm ± 1.2) to the 41-60 years group (13.9cm ± 0.9), and remained unchanged for the above 60 years group. The CTR in males increased from the 21-40 years group (46.4% ± 4.0) to the 41-60 years group (47.2% ± 3.7) and increased again minimally in the above 60 years group (47.4% ± 4.8). The CTR in females also increased from the 21-40 years group (46.4% ± 4.5) to the above 60 years group (51.7% ± 3.7). The TTD increased minimally from the 21-40 years group to the 41-60 years group, and the decreased slightly in the above 60 years group for both males and females.

**Table 1:** Mean and standard deviation of cardiac size parameters among the various age groupings

	TCD(cm)		CTR (%)		TTD(cm)	
AGE/years	Male	Female	Male	Female	Male	Female
21-40	13.6± 1.2	12.5± 1.2	46.4± 4.0	46.4± 4.5	29.4± 2.9	27.0± 2.9
41-60	14.4± 1.1	13.9± 0.9	47.2± 3.7	50.9± 3.8	30.6± 2.2	27.4± 1.6
≥ 60	13.7± 1.2	13.9± 0.7	47.4± 4.8	51.7± 3.7	29.1± 1.6	26.9± 1.7
Average	13.8± 1.2	12.9± 1.3	46.6± 3.9	49.6± 4.8	29.7± 2.7	27.1± 2.6

Table 2 and Table 3, show the expected increase in the recorded TCD values at different CTR values of 50% and 51% in males and females respectively. In males an increase from the recorded CTR to 51% is associated with a TCD difference of 1.3cm in the 21-40 years group, and a 0.1cm increase in the 41-60 years group. No increase was observed in the above 60 years group.

**Table 2:** Expected increase in TCD value at CTR of 50% and 51% (early cardiomegaly) in males

MALES			
AGP	Average		
21-40			
CTR (%)	**46.3**	**50**	**51**
TCD (cm)	13.6	14.7	15.0
TCD-TCD' (cm)	0	1.1	1.4
41-60			
CTR (%)	**47.1**	**50**	**51**
TCD (cm)	14.4	15.3	15.6
TCD-TCD' (cm)	0	0.9	1.2
61-80			
CTR (%)	**47.1**	**50**	**51**
TCD (cm)	13.7	14.5	14.8
TCD-TCD' (cm)	0	0.8	1.1

**Table 3:** Expected increase in TCD value at CTR of 50% and 51% (early cardiomegaly) in females

FEMALES			
AGP	Average		
21-40			
CTR(%)	**46.3**	**50**	**51**
TCD(cm)	12.5	13.5	13.8
TCD-TCD'(cm)	0	1.0	1.3
41-60			
CTR(%)	**50.9**	**0**	**51**
TCD(cm)	13.9	0	14.0
TCD-TCD'(cm)	0	0	0.1
61-80			
CTR(%)	**51.7**	**0**	**0**
TCD(cm)	13.9	0	0
TCD-TCD'(cm)	0	0	0

Table 4 and Table 5 show the expected CTR values after increases in the recorded TCD values by 1 to 2 cm in males and females. In males aged 21 - 40 years, an increase in TCD of 1.5cm was associated with a CTR of 51.4%, whereas in the 41-60 years group and above 60 years group, an increase in TCD of 1.0cm was associated with a CTR of 50.3% and 50.5% respectively. Increases in TCD by 1.5cm for the 41-60 years group and above 60 years group were associated with CTR values of 52.0% and 52.2% respectively. In females, an increase in TCD of 1.0cm was associated with a CTR of 50.0%, 54.4% and 55.4% for the 21-40 years group, 41-60 years group and the above 60 years group respectively. TCD increases in females of 1.5cm were associated with CTR values of 51.9%, 56.2% and 57.2% for the 21-40 years group, 41-60 years group and the above 60 years group respectively.

**Table 4:** Expected CTR after increases in TCD by 1 to 2 cm in males

MALES				
AGP	Average	1cm	1.5cm	2cm
21-40				
TCD	*13.6*	*14.6*	*15.1*	*15.6*
TTD	29.4	29.4	29.4	29.4
CTR	**46.3**	**49.7**	**51.4**	**53.1**
41-60				
TCD	*14.4*	*15.4*	*15.9*	*16.4*
TTD	30.6	30.6	30.6	30.6
CTR	**47.1**	**50.3**	**52.0**	**53.6**
> 60				
TCD	*13.7*	*14.7*	*15.2*	*15.7*
TTD	29.1	29.1	29.1	29.1
CTR	**47.1**	**50.5**	**52.2**	**54.0**

**Table 5:** Expected CTR after increases in TCD by 1 to 2 cm in females

FEMALES				
AGP	Average	1cm	1.5cm	2cm
21-40				
TCD	*12.5*	*13.5*	*14.0*	*14.5*
TTD	27.0	27.0	27.0	27.0
CTR	**46.3**	**50.0**	**51.9**	**53.7**
41-60				
TCD	*13.9*	*14.9*	*15.4*	*15.9*
TTD	27.4	27.4	27.4	27.4
CTR	**50.7**	**54.4**	**56.2**	**58.0**
> 60				
TCD	*13.9*	*14.9*	*15.4*	*15.9*
TTD	26.9	26.9	26.9	26.9
CTR	**51.7**	**55.4**	**57.2**	**59.1**

## Discussion

The CTR and TCD are common parameters used for the evaluation of cardiomegaly in the management of heart disease. The accuracy of these measurements in determining whether or not one has cardiac disease or not can therefore not be overemphasized. The study included adults comprising 397 (39.9%) females, and 650 (62.1%) males, with an age range of 21 to 80 years. The mean age of the study was 35.1 years ± 12.7. This study had a mean TCD of 13.8cm±1.2cm for males, and 12.9cm±1.3cm for females, and a CTR of 46.6% ± 3.9 for males, and 47.8% ± 4.8 for females (Table 1).

The value of the CTR as a means of evaluating heart size in an individual would depend on a constant TTD, to which the TCD can be compared. Studies have shown significant variations of TTD and hence, the CTR is regarded as an unreliable parameter by many authors [9–11]. Notwithstanding, a CTR of greater than 50% on a PA film is considered abnormal, which normally indicates cardiac enlargement or pericardial effusion, even though correlation with left ventricular ejection fraction is known to be poor [12, 13]. The TCD is a more direct measure of cardiac size, but does not always correlate well with heart disease, as pericardial effusion may also result in its increase. The TCD is considered by some as a better parameter for assessing heart size abnormalities [1, 9]. An increase of 1.5 to 2cm on two consecutive radiographs, taken at short interval, suggests possible cardiac pathology [5, 6].

This study showed that an increase in TCD of 1cm instead of 1.5cm was significant, as it resulted in a CTR of greater than 50.0% in all but the males aged 21-40 years, where the CTR was 49.7%. A 1.5cm increase in TCD on the other hand, resulted in values of CTR ranging from 51.4% to 52.2% for men, and 51.9% to 57.2% for females. These results suggest that withholding treatment till the CTR difference reaches 1.5cm is likely to delay management and allow disease progression. The average CTR values from the study (Table 2 and Table 3) showed that the increase in TCD values for the three age groups reaching the 50.0% threshold value, ranged from 0.8cm in the 61-80 year group to 1.1cm in the 21 to 40 year group for males. For females, only the 21-40 year group had a CTR (of 46.3%) below the 50.0% threshold, and required an increase of 1cm to reach a CTR of 50.0%. A TCD increase of 1.1 to 1.4cm in males, and 0.1 to 1.3cm for female was required to reach a CTR of 51%. The 41-60, and 61-80 year groups in females had average CTR values of 50.7%, and 51.7%, and are thus likely to be classified as having cardiomegaly at the first clinical visit. The study therefore shows that cardiomegaly (CTR > 50.0) may occur with a TCD increase of less than 1.5cm on consecutive PA chest radiographs, taken at short interval.

## Conclusion

The study found that the same TCD and CTR values are not suitable in detecting cardiomegaly for all age groups and genders. The differences in CTR and TCD increases noted in the study differ from those in existing publications, and appear to influence the threshold for deciding whether an individual has cardiomegaly and possibly, cardiac disease or a pericardial effusion. In diagnosing cardiomegaly or cardiac disease therefore, clinicians may have to take into consideration the local averages or normal values of the cardiac parameters, and how they compare to available published figures.

### What is known about this topic

- A cardiothoracic ratio greater than 50% is considered abnormal and suggestive of cardiomegaly;
- Transverse cardiac diameters of 15.5cm for males and 14.5cm for females are considered as the normal upper limits respectively;
- An increase in the transverse cardiac diameter by 1.5cm on two consecutive chest radiographs taken at short intervals is considered abnormal, and a sign of cardiomegaly.

### What this study adds

- The average cardiothoracic ratio differs for different age groups and genders;
- The average transverse cardiac diameter differs for different age groups and genders; hence an increase by 1.5cm does not always translate into an abnormal cardiothoracic ratio of greater than 50%;
- The average cardiothoracic ratio for females aged 41-60years, and older than 60years is 50.7% and 51% respectively. This implies these females may be regarded as having mild cardiomegaly when they may actually be normal.

## Competing interests

The authors declare no competing interest.
